# Optimization and Experimental Investigation of 3D Printed Micro Wind Turbine Blade Made of PLA Material

**DOI:** 10.3390/ma16062508

**Published:** 2023-03-21

**Authors:** Suresh Arivalagan, Rajakumar Sappani, Robert Čep, Mahalingam Siva Kumar

**Affiliations:** 1 Department of Electrical and Electronics Engineering, Varuvan Vadivelan Institute of Technology, Tamil Nadu 636701, India; 2 Department of Mechanical Engineering, Anna University Regional Campus, Tirunelveli 627007, India; 3 Department of Machining, Assembly and Engineering Metrology, Faculty of Mechanical Engineering, VSB-Technical University of Ostrava, 708 00 Ostrava, Czech Republic; robert.cep@vsb.cz; 4 Department of Mechanical Engineering, Vel Tech Rangarajan Dr. Sagunthala R&D Institute of Science and Technology, Avadi 600062, India

**Keywords:** fusion deposition modeling, micro horizontal axis wind turbine, airfoil profile, MFO, PSO, structure analysis

## Abstract

This paper presents the design, development, and optimization of a 3D printed micro horizontal axis wind turbine blade made of PLA material. The objective of the study was to produce 100 watts of power for low-wind-speed applications. The design process involved the selection of SD7080 airfoil and the determination of the material properties of PLA and ABS. A structural analysis of the blade was carried out using ANSYS software under different wind speeds, and Taguchi’s L16 orthogonal array was used for the experiments. The deformation and equivalent stress of the PLA material were identified, and the infill percentage and wind speed velocity were optimized using the moth-flame optimization (MFO) algorithm. The results demonstrate that PLA material has better structural characteristics compared to ABS material. The optimized parameters were used to fabricate the turbine blades using the fusion deposition modeling (FDM) technique, and they were tested in a wind tunnel.

## 1. Introduction

The advancement of miniature modern technology in the fields of engineering and medicine often necessitates high-performance energy consumption, a key part of most human activities. The consumption of energy involves converting one form of energy into another, mainly the conversion of mechanical energy into electrical energy. In a thermal power plant, the main role is the conversion of chemical energy into heat in the combustor, heat into mechanical energy in the turbine, and mechanical energy into electrical energy in the generator. This satisfies the power demand of commercial and industrial electrical applications [1]. However, fossil fuels are non-renewable, too expensive, and cause environmental pollution. In recent decades, a great deal of importance has been given to renewable energy system research, even though it is seasonal or time-dependent and difficult to generate a large quantity of electricity when compared with traditional fossil-fuel generators. Renewable energy is mainly considered due to its environmental benefits and the fact that it never runs out. In recent years, the micro wind turbine, which generates power even at low wind speeds, has attracted more attention from many researchers for commercial applications, especially in remote areas. The power generated through the micro wind turbine can be used in streetlights, battery reserves, and lamp posts built on national and state highways. Over a long span of operation, the expense associated with the installation of the micro wind turbine can be acquired through the amount of electricity generated. Decentralization of power generation can be achieved by installing micro wind turbines at remote locations, and this newer technology reduces the cost of establishment and power transmission losses. The conventional manufacturing technology involves more material, labor, and machinery, and the manufacturing plant itself requires huge investment. In order to minimize the cost for manufacturing micro and small-scale wind turbines, this research work proposes using newer 3D printing technology. The material selection plays a vital role in the design process of a micro wind turbine. The mass of the micro wind turbine depends on the infill condition of the geometry. It is understood that less infill reduces the mass, and more infill increases the mass. However, an optimum infill condition is required to achieve better performance and a reduced cost. Based on the literature, studies were performed on the structural analysis of micro wind turbines by using experimental and numerical techniques, particularly at low wind speeds. The wind turbine blade stability and life were predicted using structural analysis. The location experiencing the highest stress intensity was identified by additional structural analysis, using the finite element method. Optimization techniques such as the moth-flame optimization (MFO) algorithm helped to predict the optimal operating conditions and design parameters. The effectiveness of the MFO algorithm was proven by comparing it with particle swarm optimization (PSO). The literature review on wind turbine blades and 3D printing is presented in this section. The problem statement and methodology are reported in Section 2 and Section 3, respectively. The detailed results and discussion are presented in Section 4.

### 1.1. Wind Turbine Blade

A structural analysis of the wind turbine blade with respect to various design factors was performed by various researchers [2,3,4,5,6,7]. Using the finite element method, Park [2] compared the structural stability of a flax–epoxy composite blade with a glass–epoxy composite blade and reported the flax–epoxy composite blade performed better in terms of safety and structural stability. The significance of chord length and twist angle on both structural and aerodynamic characteristics is reported in [3]. Chen and Kam [4] presented an incremental loading procedure to study the progressive failure of a small, composite sandwich wind blade. Ullah et al. [5] reported that the higher stress level in the shear web can be minimized by increasing the number of biaxial plies, optimizing the blade geometry, and changing the position of shear webs, which have a profound effect on the overall performance and structural integrity of the blade. A structural stress analysis of the wind load at an extreme wind speed was reported by Wu and Young [6]. Through a fluid–structure interaction study, blade deformations and stresses were examined by Lipian et al. [7], and the influence of blade deformation on rotor performance was reported.

Mamouri et al. [8] focused on dynamic stall in offshore wind turbine blades and used the entropy generation rate as a tool for analysis and design, showing that some airfoils have a lower drag coefficient. In another work, Mamouri et al. [9] examined the entropy generation of three offshore wind turbine airfoils at various angles of attack, revealing that flow separation has a large impact on the viscous entropy generation rate and that the SD7062 airfoil has the lowest entropy generation rate at large angles of attack. Mamouri et al. [10] examined the aerodynamic coefficients of an oscillating wind turbine airfoil by conducting experiments and focusing on the effective parameters that affect the lift and drag coefficients in the blades, showing that a more suitable airfoil could be chosen based on the wind turbine’s rotation frequency. Suresh and Rajakumar [11] investigated 10 different types of airfoils for a maximum power coefficient with respect to the angle of attack and wind speed. Rahman et al. [12] studied the total deformation and maximum principal stress per unit mass with respect to three different airfoils (S811, S822, and S826) and two different materials (structural steel and aluminum alloy) for three and five blade configurations. The theoretical and experimental investigation of micro wind turbines for the low-speed region was investigated by [13]. They reported that replacing the wind-turbine-swept area with an equivalent array of micro wind turbines harnessed more wind energy. Singh and Ra [14] investigated the power coefficient with respective pitch angles and low wind velocity ranges of 3–6 m/s. They reported that the new two-bladed rotor produced more electric power compared with the baseline three-bladed rotor at the same free stream velocity. Improved performance due to multiple blade designs for a micro wind turbine at low wind speeds of 3–14 m/s is reported in [15], which addresses some innovative blade designs.

### 1.2. Three-Dimensional Printing

Complex geometric shapes can be fabricated using 3D printing with various materials. To create a multifunctional part in a traditional manufacturing system, separate traditional processes are required, whereas 3D printing can achieve production in a single step without post processing [16]. A highly complicated geometry can be manufactured using 3D printing technology, which is known as a solid freeform manufacturing technology based on layer-by-layer fabrication using computer-aided design data [17]. Additive manufacturing technology is cost-effective and highly flexible. Three-dimensional printing applications are extended to the concept modeling, functional prototyping, and digital manufacturing stages [18]. The requirements are translated into physical form within a specified time, and the functional prototype model shows the structure and performance prior to mass production [19]. Three-dimensional printing eliminates tool and mold costs, and it transforms low-volume production through mass customization [20]. Physical part stocks are replaced by digital files that can be printed [21]. Three-dimensional printing produces prototypes and functional parts through the addition of materials layer by layer, using raw materials in the form of a liquid, powder, or sheets. The model can be created in CAD software based on the original design or from a 3D scan. The model is converted into a compatible digital file called a stereolithography file (STL). The model is then exported to slicing software to generate a machine G-code, which contains the information on tool paths and makes the object layer by layer in three dimensions [22]. Various 3D printing technologies and materials are used for different applications, and the selection of technology and materials is a challenging task. The dimensional accuracy, surface finish, and post-processing requirements are different for various applications. The widest range of materials among the existing 3D printing technologies belongs to polymers, such as thermoplastic and thermoset polymers. Additive manufacturing technology has the ability to print large structures while reducing defects and improving mechanical properties [23]. There are more opportunities for 3D printing composite materials. Additive manufacturing is compatible with fiber reinforcement using polymer powders and filaments [24].

The cost, strength, filament cost, and weather resistance of the printer are all taken into consideration when choosing the print medium for a wind turbine [25]. The most popular plastics used in 3D printing are ABS (acrylonitrile butadiene styrene) and PLA (polylactic acid), and most extruders are capable of reliably extruding these materials with ease. The 3D printed wind turbine designed herein uses ABS and PLA materials, which were chosen because of their inexpensive cost, high availability, and compatibility with standard printers. The PLA’s low requirements for bed temperature are a large advantage because they allow for printing without a heated printing bed. The cost of printers without heated print beds is considerably lower than the cost of heated ones. Three-dimensional wind turbine blade production using FDM is cost-effective, depending on volume, and requires only 6–12 h to produce one one-meter blade; it takes 5–7 days to print three turbine blades [26]. The cutting edges of turbine blades are generated by 3D printing in relation to globalization. This technology will benefit from the printing of turbine blades and streamline the construction process. Engineering software aids in creating precise design specifications for the deployment of 3D printed wind turbines. A small-scale horizontal axis wind turbine was developed and produced based on an additive manufacturing background. The production of a turbine using ABS filament has a low overhead cost and can be finished in the short time of 28 h and 44 min [27].

This editorial provides a swift archetype of a wind turbine with the required parameters by providing numerical models of a wind turbine rotor [28]. A 3D printing method was used, and the product was tested in a domestic wind tunnel. The overall process was completed within one day. Smart prototype vertical axis wind turbines are designed with the highest throughput in terms of a flexible and quality design, low weight, and quick execution in printing time [29]. An efficient model was developed based on fused deposition modeling for easy access and a lower cost.

Moreover, the review article [30] elucidates the various design factors affecting the performance of a wind turbine. From the literature review, it is understood that various factors governing the power output from the turbine are the blade material, pitch, the twist angle of the blade, wind speed, number of blades, and types of airfoils [31]. In particular, blade material receives more attention due to various factors such as structural importance, the conversion of wind load into torque over the rotor, cost, and availability.

### 1.3. Moth-Flame Optimization Algorithm

Parametric optimization has been carried out by various authors using various evolutionary algorithms [32,33,34]. Among those algorithms, the recently developed moth-flame optimization (MFO) algorithm was most commonly used among researchers. The major source of inspiration for this optimizer was the transverse orientation navigation approach used by moths in nature [35]. Moths fly at night by maintaining a steady angle with the moon, which is a very effective technique for travelling vast distances in a straight line. This behavior of moths was used to effectively optimize the process parameters through the MFO algorithm. The different variants of the MFO algorithm and its applications were discussed by [36].

Yıldız and Yıldız [37] maximized the rate of profit while performing multi-tool milling operations by using the MFO algorithm. The effectiveness of the MFO algorithm was confirmed through the optimization results. Sivalingam et al. [38] used the MFO algorithm to select the optimal set of turning parameters while machining the Hastelloy X material in different machining environments. They compared the results of the MFO algorithm with the results of other evolutionary algorithms such as the genetic, grasshopper, grey wolf, and particle swarm optimization algorithms. This comparison showed that the MFO algorithm outperformed others.

## 2. Problem Statement

The aerodynamic and gravitational loads acting on the blade are considered important design factors in the installation of a wind turbine blade. The dynamic effect of these loads affects the structural rigidity of the blade. The type of materials and the material properties used for manufacturing the blade play a vital role in the structural design and cost of the micro wind blade. The fabrication of the wind blade, which works under low wind speeds for minimum power generation, is also a challenging task. The selection of the correct airfoil blade involves significant testing or simulation of the profile under different working conditions [39]. Testing a blade in a wind tunnel for different materials at various wind speeds is a very tedious job for manufacturers. Accurately measuring the deformation and stress developed during the dynamic running condition of the blade is very tough work. Three-dimensional printing is a newer technology that could reduce the cost of the production of micro and small-sized wind turbine blades and assure the structural characteristics of the wind turbine in various operating conditions. It is necessary to identify the conditions in which micro wind turbines produce more power based on the 3D printing material selection, infill condition, and wind velocity.

## 3. Methodology

### 3.1. Experiments

In this study, SD7080 airfoil was considered a suitable blade profile for producing high power in low-wind-speed applications. Based on the requirements, an aerodynamic analysis was performed using Q-Blade software on 10 selected airfoils: Aquila, BW-3, E387, FX63-137, NACA0012, NASA LS-0413, RG-15, S1223, SD7080, and SG6043 at a Reynolds number of 81,712. The lift coefficient (CL) and lift/drag ratio (c_l_/c_d_) of these airfoils were analyzed at various angles of attack at the Reynolds numbers 30,642, 40,856, 51,070, 61,284, 71,498, and 81,712. It was found that the SD7080 airfoil had the highest power coefficient relative to other airfoils at various Reynolds numbers for a tip speed ratio in the range of 5 to 8. The maximum power coefficient of 0.29 was achieved at Re = 40,856 at a tip speed ratio in the range of 5–8 for the SD7080 airfoil [11].

From the numerical simulation, it was concluded that SD7080 was the most suitable airfoil. Two different materials, acrylonitrile butadiene styrene (ABS) and polylactic acid (PLA), were selected for the 3D printing manufacture of the wind turbine blades. The properties of the blade, such as the density, Young’s modulus, and Poisson ratio for each material presented in Table 1, were obtained using the Optimatter tool by varying the infill value (IF) from 10% to 100%.

**Figure 1:**
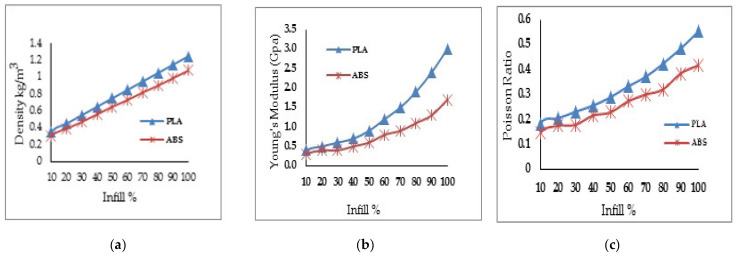
Properties of PLA and ABS materials at various IF values for (**a**) density; (**b**) Young’s modulus; (**c**) Poisson’s ratio.

Figure 1 represents the relationship between the properties and infill percentage of the PLA and ABS materials. It is inferred from Figure 1a–c that the density, Young’s modulus, and Poisson ratio values increased with an increasing infill percentage. Furthermore, for the same infill percentage, the density, Young’s modulus, and Poisson ratio in the PL material were higher than in the ABS material.

**Figure 2:**
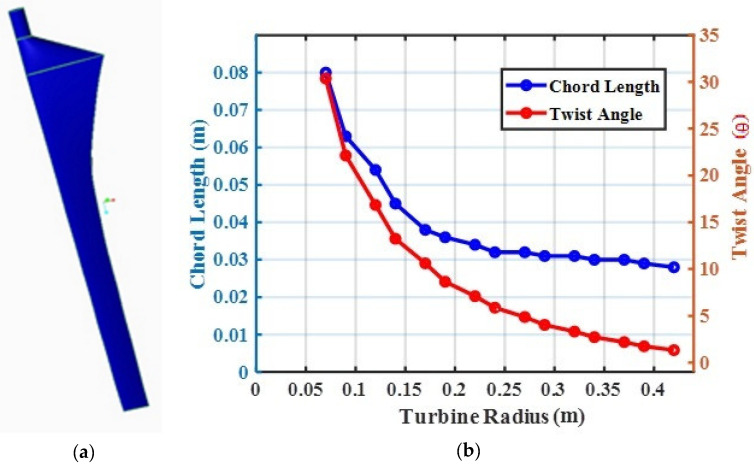
Geometry of blade profile: (**a**) 3D profile; (**b**) relationship between turbine radius and chord length and twist angle.

The blade geometry was modeled using CREO 3.0 software, which is shown in Figure 2. The specifications of the micro wind turbine blade are presented in Table 2. The mechanical properties of the blade, such as the deformation (DN) and stress (SS) values, were considered response values and measured by simulating the blade under various wind speeds (WSs), from 2 to 14 m/s, using ANSYS 15 software. The blade material type (MT), infill percentage (IF), and wind speed (WS) were considered parameters and varied at different levels, as per the value shown in Table 3. It was proposed to have a mixed-level design for experimentation. A material type with two levels, four levels of infill percentage, and four levels of wind speed were considered.

### 3.2. Finite Element Analysis

The micro wind turbine blade was designed for 100 W of power output. The design parameters and their values are presented in Table 2. The modeling of the micro wind turbine blades was performed in CREO software and exported to ANSYS software for finite element analysis. The finite element analysis (FEA) was been carried out on the wind turbine blade under various wind speeds to identify the structural characteristics, such as deformation and stress intensity. There are three stages of finite element analysis: pre-processing, solution, and post-processing. In the pre-processing stage, the elements, materials, and meshing are selected. In the solution stage, the boundary conditions, loading, and method of solving the problem are selected, and the results can be obtained in the post-processing stage.

The wind turbine blade model was created in CREO software based on the data provided in Table 2. Material properties were assigned to the blade geometry based on the data presented in Table 1. The tetrahedral element was chosen for the blade geometry meshing. Flap-wise loading on the wind turbine blade was considered for this finite element analysis. The flap-wise loading was parallel to the axis of the rotor; therefore, the axial force acting on the wind turbine blade was calculated. The wind turbine blade was designed to achieve a uniform angle of attack from root to tip, and the lift coefficient was assumed to be constant from root to tip. The axial force was directly proportional to the radius of the wind turbine blade. The axial force was at a maximum at the tip and a minimum at the root. The maximum axial force was considered for the structural analysis of the blade. The maximum axial load for the particular wind velocity on the surface of the wind turbine blade was assumed. The axial load was calculated from the wind speed using Equation (1).

(1) $$Fx=\left(\left(\frac{\pi }{9}\right)\rho D^{2}V^{2}\right)/A$$

where *F_x_* represents the pressure load (N/m^2^ or Pa); ρ represents the air density; *D* represents the blade diameter, m; *V* represents the wind velocity, m/s; area of the blade, m^2^.

The axial load was calculated from the Equation (1) and is presented in Table 4 for a wind speed of 1–15 m/s.

**Figure 3:**
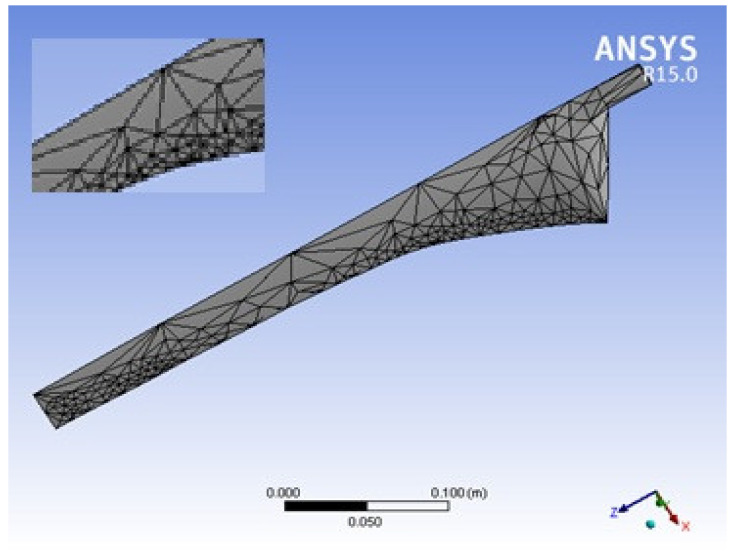
Blade meshing.

A grid-independent analysis was performed for the blade geometry, using simulation results that were adequately grid-independent [40]. The tetrahedral meshing of the blade was generated and is shown in Figure 3. Initially, the mesh was generated with 354 elements and 750 nodes. A finer mesh was then created, and a grid-independent analysis was performed for elements 520, 605, 714, and 802. The consistency of the results was identified using a grid-independent test. The deformation and stress intensity were consistent at 605 elements. The results of the grid-independent test are presented in Table 5. When the mesh elements were greater than 605, the deformation was unchanged at 0.000879 m. The results of the finite element analysis were also validated with the results of the theoretical analysis.

**Figure 4:**
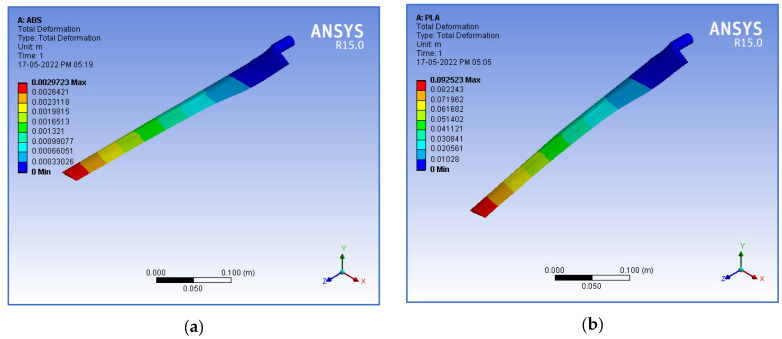

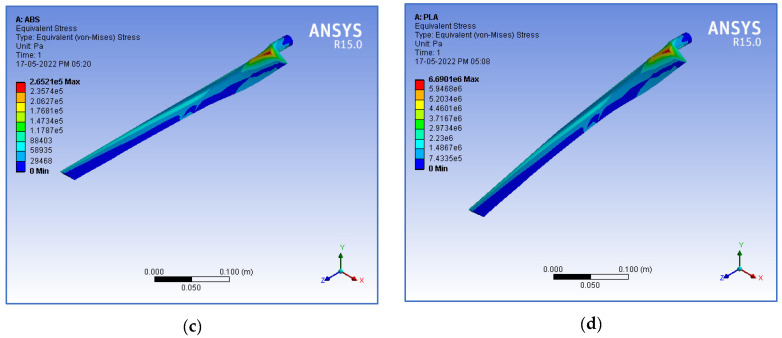
Response values from simulated results. Deformation (DN) for (**a**) ABS; (**b**) PLA. Stress intensity (SS), in Pa, for (**c**) ABS; (**d**) PLA.

The structural performance of the blade material is described by the deformation and stress intensity. Since the load is applied in the clockwise direction, the blade tends to deform in the axial direction, and tensile stress develops in the region of the hub. The boundary conditions were applied as follows: the hub of the blade was fixed, i.e., rotation and translation were arrested at the hub. Flap-wise loading was applied on the surface of the wind turbine blade. The flap-wise loading was varied at various wind velocities, as shown in Table 4. A static structural analysis was performed for a wind velocity ranging from 1 to 15 m/s and for various infill percentages from 10 to 100%. The deformation in the axial direction and the stress intensity at the wind velocity varied from 1–15 m/s, and various infill percentages from 10–100% were identified for the PLA and ABS materials. Figure 4 shows sample FEA simulation results in which the simulation outputs, such as the deformation (DN) and stress intensity (SS) developed on the blade, were considered measured response values.

### 3.3. Experimental Design and Measurements

In the present work, a mixed-level experimental design was proposed with two types of material, four levels of infill percentage, and four levels of wind speed, as shown in Table 3. Taguchi’s experimental design, which was based on the L16 orthogonal array, was selected for various combinations of parameters. The combinations of various parameters for different runs are shown in Table 6.

### 3.4. Multiple Linear Regression Models (MLRM)

**Figure 5:**
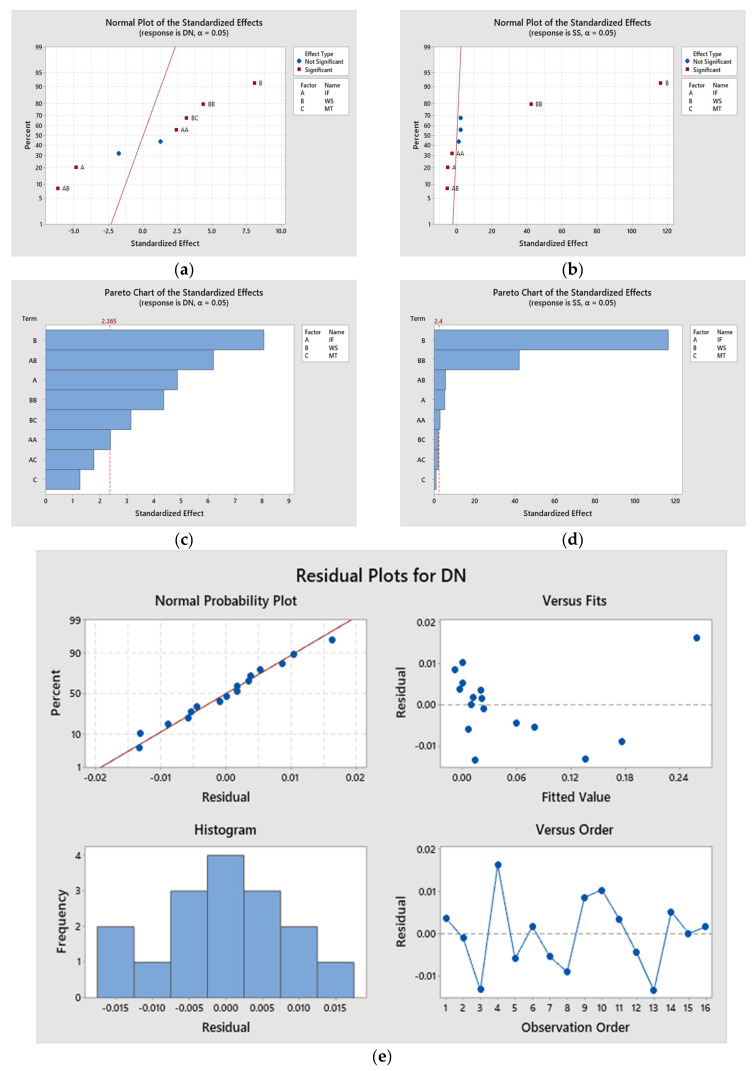

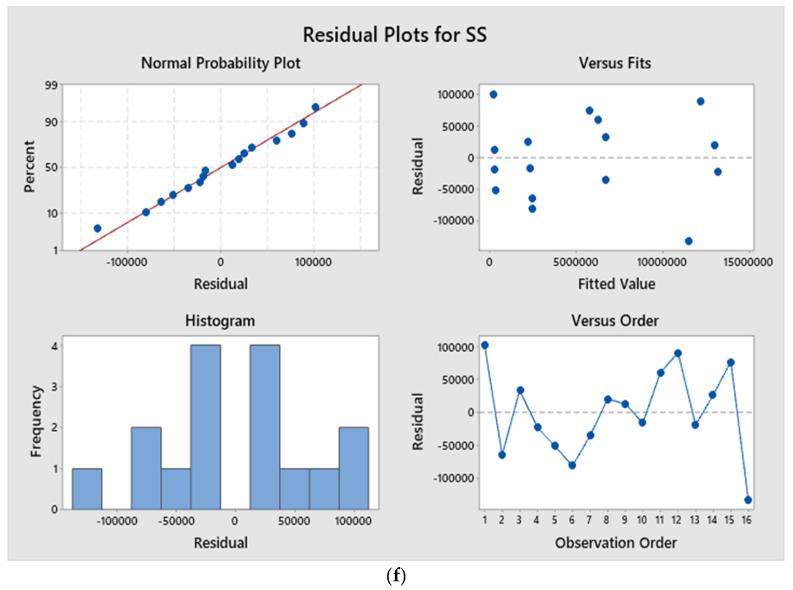
Statistical analysis of response values. (**a**) Normal plot for response DN; (**b**) normal plot for response SS; (**c**) Pareto plot for response DN; (**d**) Pareto plot for response SS; (**e**) residual plot for response DN; (**f**) residual plot for response SS.

To optimize the process parameters, it is necessary to establish MLRM equations for the experimental data. Minitab 19 software was used to develop the MLRM equations for the response values. The full quadratic model was selected to develop the MLRM equations. Equations (2) and (3) represent the full quadratic models for MLRM equations for both the deformation and stress values. The *p*-value of the model and the linear, square, and two-way interactions presented in Table 7 are all less than 0.05, indicating the statistical significance of the parameters on the response values. It is understood that R2 values greater than 95% for the MLRM equations in the responses presented in Table 8 indicate the accuracy of the model established for optimization. It is understood from Figure 5a,c that except for the material type and the interaction between the infill percentage and material type, all other factors and their interactions play a role in blade deformation. Similarly, in Figure 5b,d, the interactions between the material type and infill percentage and the material type and the wind velocity do not influence the stress exerted on the blade. Figure 5e,f indicate that the response values obtained from the experiments were normally distributed. Hence, the experiments were validated.

(2) $$\mathrm{SS}=164,817+6730\;\mathrm{IF}+49,656\;\mathrm{WS}-507,426\;\mathrm{MT}-78.3{\;\mathrm{IF}}^{2}+63,027{\;\mathrm{WS}}^{2}-1508\;\mathrm{IF}*\mathrm{WS}+5106\;\mathrm{IF}*\mathrm{MT}+38,326\;\mathrm{WS}*\mathrm{MT}$$

(3) $$\mathrm{DN}=-0.002+0.000584\;\mathrm{IF}-0.00083\;\mathrm{WS}-0.0069\;\mathrm{MT}+0.000008{\;\mathrm{IF}}^{2}+0.000608{\;\mathrm{WS}}^{2}-0.000177\;\mathrm{IF}*\mathrm{WS}-0.000478\;\mathrm{IF}*\mathrm{MT}+0.00582\;\mathrm{WS}*\mathrm{MT}$$

### 3.5. Optimization of Process Parameters

In this work, the process parameters were optimized using two different optimization techniques, namely the particle swarm optimization (PSO) and moth-flame optimization (MFO) algorithms. The normalization method expressed in Equations (4) and (5) was used to convert the DN and SS response values into between zero and one, which are on different scales. In this work, it was projected that the weights for DN and SS during normalization were taken as 25% and 75%, respectively. Equation (6) represents the objective function (NV).

(4) $$n({\mathrm{DN}}_{i})=\frac{{\mathrm{DN}}_{i}-\min_{i=1}^{rn}\left({\mathrm{DN}}_{i}\right)}{\max_{i=1}^{rn}\left({\mathrm{DN}}_{i}\right)-\min_{i=1}^{rn}\left({\mathrm{DN}}_{i}\right)}$$

(5) $$n({\mathrm{SS}}_{i})=\frac{{\mathrm{SS}}_{i}-\min_{i=1}^{rn}\left({\mathrm{SS}}_{i}\right)}{\max_{i=1}^{rn}\left({\mathrm{SS}}_{i}\right)-\min_{i=1}^{rn}\left({\mathrm{SS}}_{i}\right)}$$

(6) $${\mathrm{NV}}_{i}=0.25*n\left({\mathrm{DN}}_{i}\right)+0.75*n\left({\mathrm{SS}}_{i}\right)$$

### 3.6. Optimization Algorithms

In this work, the MFO algorithm was implemented to optimize the parameters while simultaneously minimizing both the deformation and stress developed on the blade. The pseudocode of the MFO algorithm is presented below, under Algorithm 1. The MFO parameters used in this paper are tabulated in Table 9. Due to the following advantages, the MFO algorithm was selected when compared to other recently developed algorithms:

- It provides very quick convergence at a very initial stage by switching from exploration to exploitation, which leads to an increase in the efficiency of the MFO for applications such as classifications when a quick solution is needed;
- The exploitation characteristics of the moths further reduce the flame number as a function of the iteration count;
- Simplicity, speed in searching, and simple hybridization with other algorithms.

**Algorithm 1:** Moth-Flame Optimization (MFO)
Initialize the parameters for Moth-flame
Initialize Moth position Mi randomly
For each *i* = 1:*n* do
Calculate the fitness function *fi*
End For
While (*iteration* ≤ *nitr*) do
  Update the position of *Mi*
  Calculate the no. of flames
  Evaluate the fitness function *fi*
If (*iteration* == 1) then
  *F* = *sort (M)*
   *OF* = *sort (OM)*
Else
   *F* = *sort (Mt* − 1, *Mt)*
   *OF* = *sort (Mt* − 1, *Mt)*
End if
For each *i* = 1:*n* do
For each *j* = 1:*d* do
   Update the values of *r* and *t*
   Calculate the value of *D* w.r.t. corresponding Moth
   Update *M(i,j)* w.r.t. corresponding Moth
End For
End For
End While
Print the best solution


The PSO algorithm was also used to test the performance of the MFO algorithm, and its parameters are listed in Table 9. The pseudocode of the PSO algorithm is given above, under Algorithm 2. The efficiency of the MFO was proven by comparing the results obtained using the PSO algorithm.

**Algorithm 2:** Particle Swarm Optimization (PSO)*P* = Particle Initialization ();
For *i* = 1 to *nitr*
 For each particle *p* in *P* do
  *fp* = *f(p)*;
  If *fp* is better than *f(pBest)*;
   *pBest* = *p*;
  end
 end
 *gBest* = best *p* in *P*
 For each particle *p* in *P* do
  *v* = *v* + *c1* ∗ *rand* ∗ *(pBest − p)* + *C2* ∗ *rand* ∗ *(gBest − p)*;
  *p* = *p* + *v*;
 end
end


## 4. Results and Discussions

### 4.1. Parameter’s Effect on Response Values

**Figure 6:**
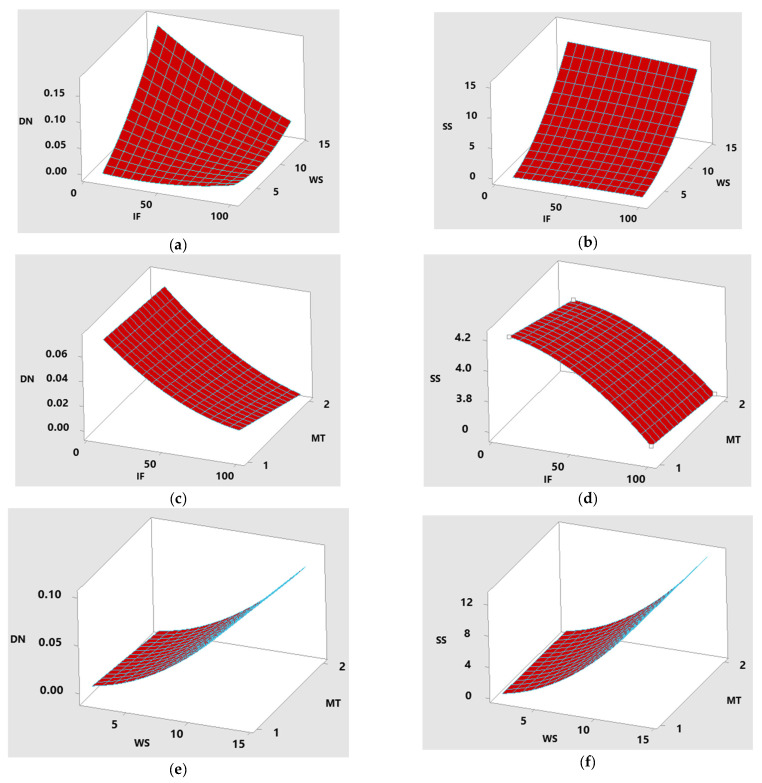
Effect of parameters on response values. (**a**) Surface plot of DN—hold value of MT: 1.5; (**b**) surface plot of SS—hold value of MT: 1.5; (**c**) surface plot of DN—hold value of WS: 8; (**d**) surface plot of SS—hold value of WS: 8; (**e**) surface plot of DN—hold value of IF: 155; (**f**) surface plot of SS—hold value of IF: 55.

Figure 6 illustrates the surface plots of various parameters concerning the response values of DN and SS. To construct the surface plots for demonstration purposes, the hold values of material type, infill percentage, and wind speed were assumed to be 1.5, 55, and 8, respectively. It is understood from Figure 6a,b that the interaction effect between IF and WS will increase both the deformation and stress on the blade while increasing the IF and WS values in both the PLA and ABS materials. When the IF value in PLA exceeds 50%, the deformation is less than that of ABS. In the ABS material, the stress value is high compared to the PLA material, which has less than 50% of the IF value. These effects are shown in Figure 6c,d. For speeds of less than 5 m/s WS, both deformation and stress are lower in the PLA material compared to the ABS material. This is understood from Figure 6e,f.

### 4.2. Optimization of Response Values Using MFO and PSO

The optimized parameters for the simultaneous minimization of deformation and stress were obtained by implementing the MFO algorithm. The coding of the MFO algorithm was developed using MATLAB programming. For each run of the program, an iteration number of 100 was assumed to be the stopping criteria. The Pareto optimal fronts obtained in each run were converted from multiple objectives into a single objective, using the normalization method [41]. The response values and their parameters corresponding to the minimum objective value were considered the optimal response values and parameters. The program had to be run 12 times, and the best of each run was recorded. Table 10 and Table 11 represent the same for the MFO and PSO algorithms, respectively. The response values of 12 runs were again converted to a single objective using the same normalization technique, and they are presented in Table 10 and Table 11 for the MFO and PSO algorithms, respectively.

**Figure 7:**
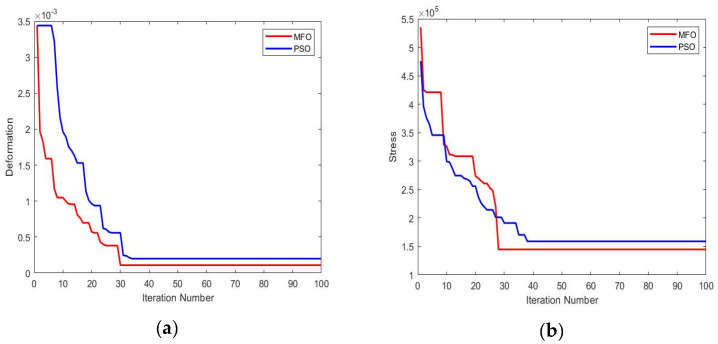
Convergence plots for (**a**) response DN; (**b**) response SS.

Figure 7 illustrates the convergence plot of the response values DN and SS. It is inferred from the figure that both DN and SS values are converged in a smaller number of iterations in the MFO algorithm than the PSO algorithm. Quick convergence by changing exploration to exploitation in the initial stage of the MFO produced optimal results in the minimum number of iterations. Apart from the convergence, the smaller number of parameters, simplicity, flexibility, scalability, speed in searching, and the lack of derivation information required in the initial phase were some of the other valid points that increased the efficiency of the MFO algorithm compared to the PSO algorithm.

### 4.3. Statistical Analysis of Optimization Results

**Figure 8:**
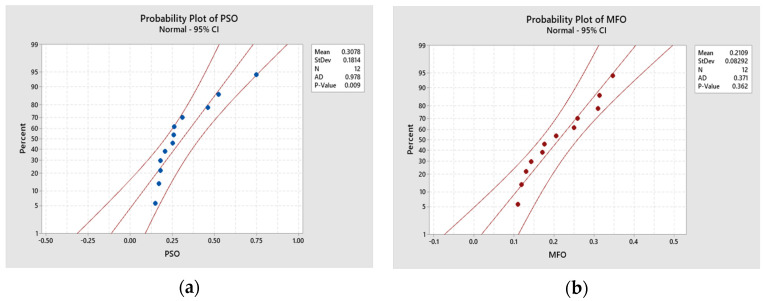

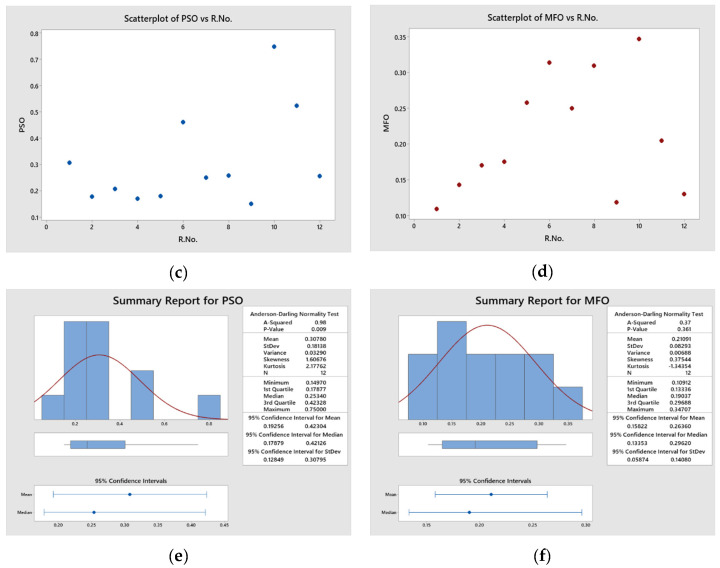
Statistical analysis and normality test for 12 runs. (**a**) Probability plot for PSO; (**b**) probability plot for MFO; (**c**) scatter plot for PSO; (**d**) scatter plot for MFO; (**e**) statistical details for PSO; (**f**) statistical details for MFO.

In this study, both the PSO and MFO algorithms coded in MATLAB were executed 12 times, i.e., 12 runs. In each run, 100 iterations were taken as the stopping criterion. Since both the responses considered in this study were of a minimization nature, the dual objective values were converted into a single objective using the normalization method. The statistical analysis of these values is presented in Figure 8. Minitab software, version 19, was used to establish probability, scatter, normal probability, and distribution plots. The probability values in the MFO and PSO algorithms shown in Figure 8a,b are greater than 0.005, and there is no pattern followed in Figure 8c,d. These results indicate that the output values obtained from the algorithms are from the population of normal distribution. Moreover, in Figure 8e,f, the mean, median, and standard deviation values for both algorithms fall under the 95% confidence interval, and the *p* values are above 0.005, indicating that the results obtained by using the algorithms are normally distributed. Therefore, the parameters and their corresponding responses are acceptable.

**Figure 9:**
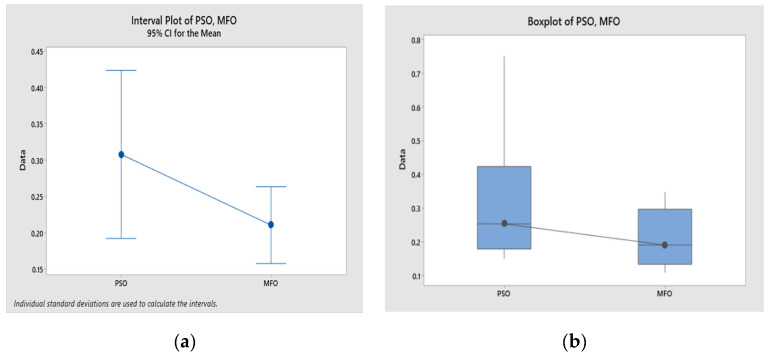
Statistical results of paired *t*-test. (**a**) Interval plot of PSO and MFO; (**b**) boxplot of PSO and MFO.

The significant difference between the results obtained using the PSO and MFO algorithms was confirmed with paired *t*-test results of descriptive statistics, an estimation of paired difference, and the test values shown in Table 12, Table 13 and Table 14, respectively. It was assumed that there was no difference in mean value (the null hypothesis). This test was run using Minitab 19 software, and the results are shown in Figure 9. The *p*-value shown in Figure 9 from the paired *t*-test is 0.05, rejecting the null hypothesis. This ensures that the results obtained using the PSO and MFO algorithms are significantly different.

### 4.4. Non-Parametric Test for Statistical Comparison of Algorithms

To compare the efficiency of the proposed MFO algorithm with the PSO algorithm, a statistical comparison was carried out using the non-parametric Friedman test, implemented by [42,43,44].

#### Friedman Test

This test was carried out to find the significant difference between the algorithms’ results in a similar way to the testing of a two-way analysis of variance. In this test, equal population medians are assumed to be a null hypothesis. This test was carried out using the “friedman()” function available in the MATLAB 2021a software. The statistical table, as the output of the function, is shown in Table 15. From the *p*-value of 0.0209 in Table 15, it is understood that the null hypothesis is rejected, and the results are significant. The mean rank values of the MFO and PSO algorithms were obtained at 1.1667 and 1.833, respectively, using the above MATLAB function. It is understood that the MFO algorithm, which had a low mean rank value, was the best option compared to the PSO algorithm, proving that the MFO algorithm outperformed the PSO algorithm.

The optimal values of MT, IF, and WS for simultaneously minimizing the DN and SS values were evaluated using the above-mentioned algorithms, and the results are presented in Table 16. The results show that the MFO algorithm outperformed the PSO algorithm.

### 4.5. Confirmation of Experiment Results

**Figure 10:**
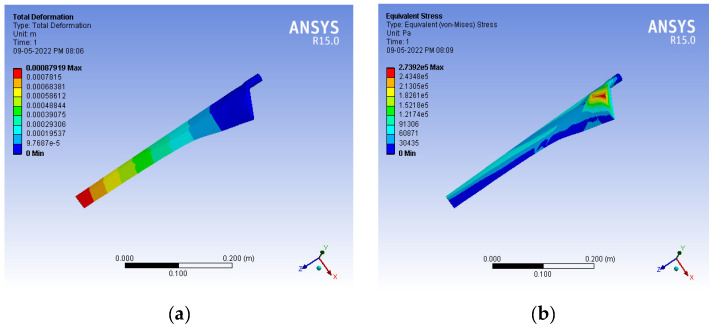
Confirmation experimental results for (**a**) response DN; (**b**) response SS.

A confirmation experiment was carried out for the parameter settings of IF and WS at 79.77% and 2.015 m/s, respectively, for the PLA material. The experimental values of DN and SS are listed in Table 17, which are 3.06% and 0.68% less than the predicted values, respectively. This demonstrates that the optimum parameter and response values obtained using the MFO algorithms are acceptable. To support this, simulation results are also reported in Figure 10.

### 4.6. Fabrication and Testing

The fused deposition modeling (FDM) method of additive manufacturing technology was used for blade fabrication. It is closest to “bottom-up’ manufacturing in which a structure can be built according to a complicated design shape using a “layer-by-layer” approach rather than casting or forming, such as inflating or machining [45]. FDM is based on the fact that the thermoplastic materials in the fiber are melted and bonded to the previous layer by melting the formed layer. It is based on prefabricating a contemporary layer on the top layer by melting the thermoplastic material in the fiber [46].

Using CREO software, a 100 W 3D CAD model of the micro wind blade with dimensions of 0.415 m blade radius, 0.0802 m root chord length, and 0.0280 m root tip length was created. The modeled geometry was converted into an STL file by considering the optimal infill percentage of 79.77, obtained from the MFO algorithm, which was processed further using the flash print software “Slicer” [47] to fabricate the blade using the 3D printing fusion deposition method. The blade was then taken to be tested in the wind tunnel. The FDM 3D printer used a PLA filament with a thickness of 1.75 mm. The 3D printer’s extruder supplied the PLA material at 220 °C and formed the geometry of the blade layer by layer with a thickness of 0.1 to 0.4 mm. The total numbers of layers was 802, and time taken to complete the fabrication of blade was 95 min.

The output voltage, current, and speed of the rotor were considered measuring parameters for assessing the power developed by the turbine.

**Figure 11:**
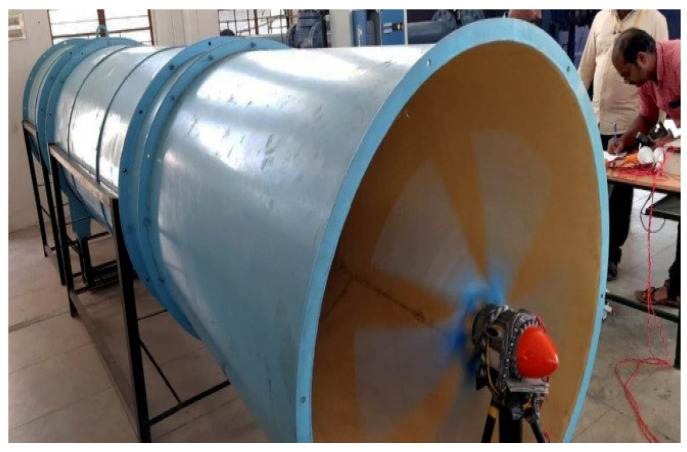
Experimental setup of micro wind turbine.

The cross-section diameter of the wind tunnel nozzle was 113 cm, and its center was 87 cm above the ground. The capacity of the fan motor was 5.5 hp with three-phase AC, and its rotational speed was 1440 rpm. The wind turbine blades were attached to the hub with a permanent magnet generator, and this rotor–hub assembly was kept in front of the nozzle (Figure 11).

To measure the wind speed, a digital anemometer with a resolution of 0.1 m/s and an accuracy of 2% + 0.1 m/s, the Lutron AM-4201 model (0.4–30 m/s), was used. A Lutron DT2234C tachometer, a non-contact laser type, was used to measure the wind turbine speed. A DT830D LCD display digital multimeter was connected to the load to measure the voltage and current. Two Wibro Garnet base B22-50w led bulbs were considered a load on the wind turbine. 

The 100 W, five-blade micro wind turbine model with an SD7080 airfoil was tested at different wind speeds. The wind velocity controller controlled the velocity inside the wind tunnel. Due to an aeroelastic phenomenon, when the wind speed was raised beyond 13 m/s, the vibration was sensed during the experiment. Therefore, the wind turbine was not evaluated for speeds greater than 12 m/s. The power output of the wind turbine at various wind speeds was observed, and wat is found that at 12 m/s of wind velocity, the turbine produced 100 W of power output.

## 5. Conclusions

A new, efficient airfoil blade made using PLA and ABS materials was designed to work in low-wind-speed applications for a micro horizontal axis wind turbine. The micro wind turbine blade was modeled using CREO software, and a finite element analysis was performed using ANSYS software. Taguchi’s L16 orthogonal array experimental design was considered for conducting experiments under various parameter combinations. The finite element results for the deformation and stress were considered response values, and corresponding MLRM equations were established. The MFO algorithm was implemented to obtain the optimal parameters and compare their effectiveness with the PSO algorithm. A statistical analysis confirmed that the results obtained by the algorithms were from the normal distribution. A paired *t*-test and a non-parametric Friedman test proved that the results from the MFO algorithm outperformed the PSO algorithm. The finite element analysis was carried out on the blade as a confirmation experiment for the PLA material with a 79.77% IF value under a wind speed of 2.015 m/s; these values were obtained from the MFO algorithm as the optimum parameters. A deviation of less than 3% was recorded between the predicted and experimental response values. Using FDM, the turbine blades were also fabricated using the PLA material with 79.77% infill. The micro wind turbine was tested in the wind tunnel and achieved a power output of 97 W.

## Figures and Tables

**Table 1:** Blade properties.

Infill (%)	PLA			ABS		
	Density (g/m^3^)	Young’s Modulus (Gpa)	Poisson Ratio	Density (g/m^3^)	Young’s Modulus (Gpa)	Poisson Ratio
10	0.35	0.40	0.184	0.30	0.30	0.146
40	0.65	0.70	0.256	0.56	0.50	0.216
70	0.95	1.50	0.371	0.82	0.90	0.300
100	1.25	3.00	0.552	1.08	1.70	0.417

**Table 2:** Specification of micro wind turbine blade.

Parameter	Range
Power	100 W
Profile	SD7080 (9.2%)
Axis of rotation	Horizontal
No. of Blades	5
Radius of the Blade	0.415 m
Root Chord Length	0.0802 m
Tip Chord Length	0.0280 m

**Table 3:** List of Parameters and its levels.

Parameter/Levels	Level 1	Level 2	Level 3	Level 4
Infill (IF), %	10	40	70	100
Wind Speed (WS), m/s	2	6	10	14
Material Type (MT)	1 (ABS)	2 (PLA)	-	-

**Table 4:** Axial loading at various wind speeds.

Wind Speed (m/s)	Axial Load (N/m^2^)	Wind Speed (m/s)	Axial Load (N/m^2^)	Wind Speed (m/s)	Axial Load (N/m^2^)
1	12	6	446	11	1500
2	50	7	607	12	1785
3	112	8	793	13	2094
4	198	9	1004	14	2429
5	310	10	1239	15	2788

**Table 5:** Grid-independent analysis.

S. No.	Nodes	Elements	Maximum Deformation (m)
1	750	354	0.000891
2	1320	520	0.000881
3	1770	605	0.000879
4	2300	714	0.000879
5	3250	802	0.000879

**Table 6:** L16 Taguchi orthogonal array experimental values.

E. No.	IF (%)	WS (m/s)	MT	DN (mm)	SS (Pa)
1	10	2	1	0.004901	2.70 × 10^5^
2	10	6	1	0.044109	2.43 × 10^6^
3	10	10	2	0.092523	6.69 × 10^6^
4	10	14	2	0.18135	1.31 × 10^7^
5	40	2	1	0.002972	2.65 × 10^5^
6	40	6	1	0.026753	2.39 × 10^6^
7	40	10	2	0.053203	6.55 × 10^6^
8	40	14	2	0.104280	1.28 × 10^7^
9	70	2	2	0.000976	2.52 × 10^5^
10	70	6	2	0.008785	2.26 × 10^6^
11	70	10	1	0.041315	6.46 × 10^6^
12	70	14	1	0.080978	1.27 × 10^7^
13	100	2	2	0.000431	2.82 × 10^5^
14	100	6	2	0.003722	2.09 × 10^6^
15	100	10	1	0.020989	6.15 × 10^6^
16	100	14	1	0.041138	1.20 × 10^7^

**Table 7:** ANOVA for response values.

Source	DF	DN				SS			
		Adj SS	Adj MS	F-Value	*p*-Value	Adj SS	Adj MS	F-Value	*p*-Value
Model	8	0.067044	0.008380	94.08	0.000	3.49954 × 10^14^	4.37443 × 10^13^	4810.47	0.000
Linear	3	0.010788	0.003596	40.37	0.000	1.23113 × 10^14^	4.10378 × 10^13^	4512.84	0.000
IF	1	0.003344	0.003344	37.54	0.000	2.55568 × 10^11^	2.55568 × 10^11^	28.10	0.001
WS	1	0.007189	0.007189	80.70	0.000	1.22849 × 10^14^	1.22849 × 10^14^	13,509.42	0.000
MT	1	0.000256	0.000256	2.87	0.134	9,225,602,500	9,225,602,500	1.01	0.347
Square	2	0.002254	0.001127	12.65	0.005	1.63505 × 10^13^	8.17526 × 10^12^	899.02	0.000
IF ∗ IF	1	0.000738	0.000738	8.29	0.024	79383062500	79383062500	8.73	0.021
WS ∗ WS	1	0.001516	0.001516	17.02	0.004	1.62711 × 10^13^	1.62711 × 10^13^	1789.31	0.000
2-Way Interaction	3	0.005404	0.001801	20.22	0.001	3.79126 × 10^11^	1.26375 × 10^11^	13.90	0.002
IF ∗ WS	1	0.004058	0.004058	45.56	0.000	2.94578 × 10^11^	2.94578 × 10^11^	32.39	0.001
IF ∗ MT	1	0.000370	0.000370	4.16	0.081	42,243,368,056	42,243,368,056	4.65	0.068
WS ∗ MT	1	0.000976	0.000976	10.95	0.013	42,304,668,056	42,304,668,056	4.65	0.068
Error	7	0.000624	0.000089			63,654,826,389	9,093,546,627		
Total	15	0.067667				3.50018 × 10^14^			

**Table 8:** R-squared values of responses SS and DN.

Response	R-sq	R-sq (Adj)	R-sq (Pred)
SS	99.08%	98.03%	93.23%
DN	99.98%	99.96%	99.87%

**Table 9:** Parameters and their values used in PSO and MFO algorithms.

PSO Algorithm		MFO Algorithm	
Parameter	Value	Parameter	Value
Learning factors (*C*1 & *C*2)	2 & 2	Position of moth close to the flame (*t*)	−1 to −2
Inertia weight (*ω*)	0.6	Update mechanism	Logarithmic spiral
Particle size (*N*)	30	No. of moths (*N*)	30

**Table 10:** Optimum parameters and their responses with normalized values for 12 runs using the MFO algorithm.

R.No.	IF	WS	MT	DN	SS	n (DN)	n (SS)	NV
1	79.77	2.015	2	0.000907	272,262	0.22482	0.07055	0.10912
2	51.67	3.854	2	0.000102	945,789	0.35912	0.07055	0.14269
3	18.09	2.021	1	0.002426	228,835	0.64926	0.01062	0.17028
4	21.13	2.030	1	0.002269	244,637	0.60532	0.03243	0.17565
5	81.01	2.749	2	0.000111	469,993	0.00255	0.34341	0.25820
6	82.59	2.865	2	0.000389	505,187	0.08018	0.39198	0.31403
7	12.09	2.085	1	0.003682	221,139	1.00000	0.00000	0.25000
8	30.94	2.991	2	0.000105	520,275	0.00070	0.41280	0.30978
9	17.17	2.533	2	0.000849	285,557	0.20850	0.08889	0.11880
10	82.34	3.009	2	0.000119	555,351	0.00466	0.46120	0.34707
11	80.20	2.579	2	0.000114	418,488	0.00332	0.27234	0.20508
12	79.98	2.169	2	0.000700	306,622	0.16713	0.11796	0.13025

**Table 11:** Optimum parameters and their responses with normalized values for 12 runs using the PSO algorithm.

R.No.	IF	WS	MT	DN	SS	n (DN)	n (SS)	NV
1	80.15	2.089	2	0.000908	287,092	0.20025	0.13281	0.14967
2	81.35	2.527	2	0.000547	401,606	0.10914	0.37429	0.30800
3	81.84	2.165	2	0.001388	304,010	0.32136	0.16848	0.20670
4	81.58	2.034	2	0.001566	273,285	0.36627	0.10369	0.16934
5	80.29	2.436	2	0.000357	376,872	0.06094	0.32213	0.25684
6	23.76	2.716	2	0.000204	384,499	0.02233	0.33822	0.25924
7	84.44	3.402	2	0.000115	698,321	0.00000	1.00000	0.75000
8	80.27	2.190	2	0.000767	311,655	0.16457	0.18460	0.17960
9	82.34	3.009	2	0.000119	555,351	0.00089	0.69851	0.52410
10	11.03	2.110	1	0.004075	224,114	1.00000	0.00000	0.25000
11	80.72	2.152	2	0.000998	301,756	0.22294	0.16373	0.17853
12	81.92	2.886	2	0.000175	513,682	0.01503	0.61063	0.46173

**Table 12:** Descriptive statistics of paired *t*-test and CI: MFO; PSO.

Sample	N	Mean	StDev	SE Mean
MFO	12	0.2109	0.0829	0.0239
PSO	12	0.3078	0.1814	0.0524

**Table 13:** Estimation for paired difference.

Mean	StDev	SE Mean	95% CI for μ_Difference
−0.0969	0.1481	0.0427	(−0.1910, −0.0028)

**Table 14:** Hypothesis and test value.

Hypothesis	Meaning	T-Value	*p*-Value
Null hypothesis	H₀: μ_difference = 0	−2.27	0.045
Alternative hypothesis	H₁: μ_difference ≠ 0		

**Table 15:** Friedman test results.

Source	Sum Square	Degrees of Freedom	Mean Square	Chi Square Value	Probability > Chi Square Value
Columns	2.66667	1	2.66667	5.33	0.0209
Error	3.33333	11	0.30303		
Total	6	23			

**Table 16:** Optimum parameters for MFO and PSO algorithms.

Algorithm	IF	WS	MT	DN	SS	n (DN)	n (SS)	NV
MFO	79.77	2.015	2	0.000907	272,262	0	0	0
PSO	80.15	2.089	2	0.000908	287,092	1	1	1

**Table 17:** Comparison of response values between predicted and experimental values.

Parameters Setting at IF = 79.77%, WS = 2.015 and MT = PLA	DN	SS
Predicted value	0.000907	272,262
Experimental value	0.000879	273,920
Error %	3.06	−0.68

## Data Availability

The data presented in this study are available upon request through email to the corresponding author.
